# A High-Resolution View of Genome-Wide Pneumococcal Transformation

**DOI:** 10.1371/journal.ppat.1002745

**Published:** 2012-06-14

**Authors:** Nicholas J. Croucher, Simon R. Harris, Lars Barquist, Julian Parkhill, Stephen D. Bentley

**Affiliations:** Pathogen Genomics, Wellcome Trust Sanger Institute, Wellcome Trust Genome Campus, Hinxton, Cambridge, United Kingdom; University of Oxford, United Kingdom

## Abstract

Transformation is an important mechanism of microbial evolution through which bacteria have been observed to rapidly adapt in response to clinical interventions; examples include facilitating vaccine evasion and the development of penicillin resistance in the major respiratory pathogen *Streptococcus pneumoniae*. To characterise the process in detail, the genomes of 124 *S. pneumoniae* isolates produced through *in vitro* transformation were sequenced and recombination events detected. Those recombinations importing the selected marker were independent of unselected events elsewhere in the genome, the positions of which were not significantly affected by local sequence similarity between donor and recipient or mismatch repair processes. However, both types of recombinations were sometimes mosaic, with multiple non-contiguous segments originating from the same molecule of donor DNA. The lengths of the unselected events were exponentially distributed with a mean of 2.3 kb, implying that recombinations are stochastically resolved with a fixed per base probability of 4.4×10^−4^ bp^−1^. This distribution of recombination sizes, coupled with an observed under representation of large insertions within transferred sequence, suggests transformation has the potential to reduce the size of bacterial genomes, and is unlikely to act as an efficient mechanism for the uptake of accessory genomic loci.

## Author Summary

Transformation is the process by which cells take up DNA from the environment and integrate it into their genome. It was first observed in the bacterium *Streptococcus pneumoniae*, a common cause of pneumonia and meningitis. This ability has allowed *S. pneumoniae* to evolve resistance to penicillin and to change its surface antigens to evade vaccines. To characterise this process in detail, we transformed an *S. pneumoniae* strain in the laboratory and sequenced the genomes of the resultant mutants. This showed that multiple recombinations, arising from different molecules of imported DNA, could occur around the genome at the same time. Some individual imported molecules donated multiple segments of sequence into the recipient. The positions of the recombinations were not significantly affected by the level of sequence similarity between donor and recipient, as had previously been observed for transfers between species, or by the mismatch repair process, which had previously been found to inhibit the transfer of small numbers of mutations. The recombinations' lengths were exponentially distributed with a mean length of 2.3 kb. This implies that the smallest version of a genetic locus will become the most common in the bacterial population in the absence of selection for a longer alternative.

## Introduction

Genetic transformation is the process by which cells take up DNA from the environment and integrate the sequence into their genome. Over sixty bacterial species, found in both Gram positive and negative phyla, have been shown to be naturally transformable in the laboratory [1]. These include important human pathogens such as *Streptococcus pneumoniae* (the pneumococcus), the bacterium in which this phenomenon was first observed [2]. The discovery that the ‘transforming principle’ involved was DNA proved crucial in establishing the chemical nature of the hereditary material [3].

Pneumococci have a dedicated system for the acquisition of DNA from the environment. The double-stranded DNA (dsDNA) first contacts a pseudopilus [4], [5], then progresses to the uptake pore complex [6]. One strand is degraded [7], [8] while the other is cleaved into fragments with a median size of ∼6.6 kb as it enters the cell [8], [9]. Proteins such as RecA and DprA are loaded onto the ssDNA once it is in the cytosol, forming a nucleoprotein filament capable of invading the host chromosome at regions of similar sequence [10], [11]. This permits the integration of the acquired DNA, and hence the horizontal transfer of polymorphisms.

*In vitro* studies of this process using single polymorphism markers have the identified transversion mutations A•T↔C•G and C•G↔G•C as markers transferred via transformation with a high efficiency, the transversion A•T↔T•A as having an intermediate efficiency, while transitions acted as low efficiency markers [12]–[14], although some inconsistencies, ascribed to neighbouring sequence context, were identified. These differences represent the efficiency with which these mutations are processed by the mismatch repair (MMR) system [15]–[21]. Deletions of 3 bp or shorter also act as low efficiency markers that are subject to MMR [14], [22], [23], but those of 5 bp or longer tend to be transferred at a relatively higher rate due to less effective repair by MMR [12]–[14], [23]–[25].

However, the effects of MMR are abrogated by high numbers (more than ∼150) of imported polymorphisms saturating the repair mechanism [26]: were this not the case, many of the recombinations observed to occur both *in vitro* and would be impossible [27]. Nevertheless, an inverse linear *in vivo*relationship is observed between the mean level of sequence divergence and the logarithm of the frequency of recombination events, implying higher densities of polymorphisms also constitute a significant barrier to the exchange of sequence between bacteria [27]–[29]. This has been suggested to be the consequence of the requirement for a minimum threshold length of perfect sequence identity between the donor strand and recipient duplex (a ‘Minimal Efficiently Processed Segment’, or MEPS) at each end of a recombination to allow a crossover to occur [30]. Based on the changing frequency of transfer with donor sequences of different levels of divergence from the recipient, the minimum summed length of the two MEPS flanking *S. pneumoniae* recombinations was estimated to be 27 bp [27].

The lengths of recombination events themselves have been estimated using several methods. The transfer of multiple polymorphic markers simultaneously found the mean size of recombinations to be around 2 kb [24]. Subsequent experiments investigating the integration of isotopically labeled DNA suggested a mean size between 3 and 6 kb [31], [32], while a mean of ∼4.4 kb was inferred using species-wide multilocus sequence typing data [33]. Similar sized events were observed through sequencing *pbp* genes transformed using plasmid DNA *in vitro* [34]. More recent estimates have been produced by sequencing the genomes of closely related *S. pneumoniae* isolates: a recent study of the PMEN1 lineage suggested a mean of 6.3 kb [35], while putative transfers between co-colonising strains indicated a mean of between 6.9 and 28 kb [36].

Here we describe work focussed on recombinations occurring at the *S. pneumoniae* capsule biosynthesis (*cps*) locus. Recombinations affecting this region that alter the capsule type of *S. pneumoniae* have been observed to lead to evasion of anti-pneumococcal polysaccharide conjugate vaccines, which target only a subset of capsule types [35], [37]. Furthermore, *pbp2X* and *pbp1A*, two genes encoding penicillin-binding proteins crucial in determining pneumococcal β lactam susceptibility, closely flank the *cps* locus. Hence long recombination events can simultaneously lead to both vaccine escape and the acquisition of penicillin resistance [37], [38]. However, such large events may be disfavoured when *cps* loci are transferred from a penicillin-sensitive donor to a resistant recipient [35]. In order to study the nature of these events, and other recombinations around the chromosome, we applied next generation sequencing to selected transformants produced under controlled laboratory conditions.

## Results

### Recombination size limits linkage between loci

**Figure 1:**
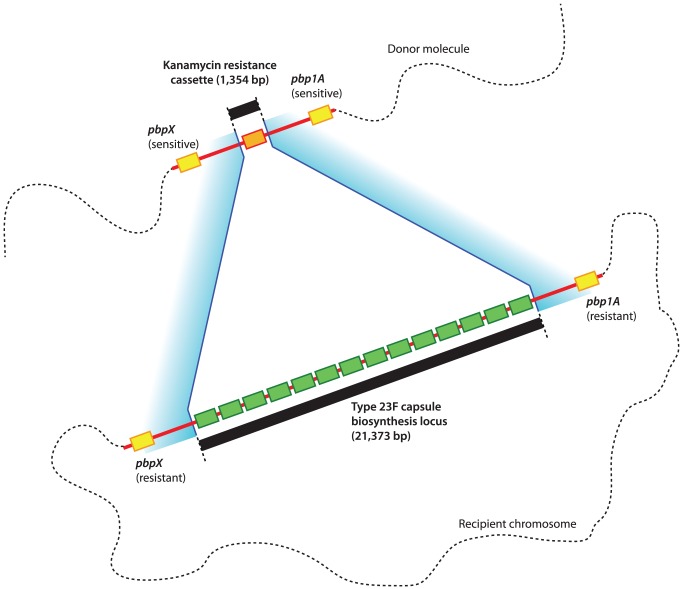
Cartoon representing the structure of the selected transformations around the pneumococcal *cps* locus. The kanamycin resistance cassette (orange) on the donor DNA molecule is flanked by *pbp* genes (yellow), both of which are β lactam-sensitive alleles. The equivalent locus in the recipient chromosome is occupied by the much longer type 23F capsule biosynthesis cluster (represented by a series green boxes to indicate the relatively large number of genes comprising this cluster), which is flanked by *pbp* alleles that confer β lactam resistance on the host cell. The selected transformations involve in the exchange of DNA from the donor into the recipient, with boundaries in the regions shaded blue.

The first experiment involved the transformation of an isolate of *S. pneumoniae* ATCC 700669, a serotype 23F strain of the penicillin-resistant PMEN1 lineage [39] (henceforth referred to as 23F-R), with genomic DNA from an acapsular derivative of the penicillin-sensitive *S. pneumoniae* TIGR4 strain, which has a kanamycin resistance marker in place of a capsule biosynthesis (*cps*) locus [40] (henceforth referred to as TIGR4Δ*cps*) (Figure 1). Multiple transformations were performed using a concentration of either 5 ng mL^−1^ or 500 ng mL^−1^ of donor genomic DNA. Recombinations affecting the *cps* locus were detected either through selection with kanamycin alone, or kanamycin supplemented with ampicillin. This latter condition was used to test whether the transfer of capsule loci from the β lactam-sensitive donor to the resistant recipient may be inhibited by selection against any co-transfer of β lactam-sensitive PBPs [35].

With selection on just kanamycin, the transformation with the lower concentration of DNA, relative to the higher concentration, produced 38-fold fewer transformants (Wilcoxon test, *p* = 0.024; Table S1), suggesting that the availability of the marker was limiting the frequency of transformation. However, dual selection with ampicillin caused a small, but non-significant, decrease in the number of observed transformants. Therefore, selection for β lactam resistance does not appear to significantly inhibit exchange with penicillin-sensitive lineages at the *cps* locus, instead suggesting a strong limitation on the size of recombination events reducing the impact of linkage between genes. To test this hypothesis, 21 isolates from each of the four examined conditions (low and high DNA concentration, and with and without ampicillin selection) and a sample of the donor DNA were sequenced using the Illumina platform.

### Multiple sequence imports within individual cells

Alignment of the complete genome sequences of the donor and recipient strains identified 21,541 base substitutions (a mean density of one per 96 bp of sequence aligned between the donor and recipient), 579 insertions relative to 23F-R (1 bp–14,153 bp in size) and 477 insertions relative to TIGR4Δ*cps* (1 bp–76,847 bp in size). The apparent absence of a strong discernable population structure among pneumococci [41], other than the association of very closely related isolates, suggests that this pairing should be broadly representative of interactions between different genotypes across the species. Using the criteria described by Harris *et al.* [42], Illumina sequence reads generated from the donor DNA identified 17,534 SNPs when aligned to the recipient sequence, of which 385 appeared to be false positives that did not correspond to polymorphisms identified through the whole genome alignment. These positions were excluded from subsequent analyses. Similarly, sequence data from the 84 transformants identified 2,312 polymorphic sites, of which just 59 did not correspond to polymorphisms transferred from the donor. Six of these sites were false positives also identified using reads from the donor DNA, whereas many of the others appear likely to be the consequence of *de novo* point mutations or intragenomic recombinations (examples involving an IS element and the repetitive protein *pclA* were observed). It is possible that this latter phenomenon may have been promoted by the upregulation of the recombination machinery, such as *recA*, during competence [43], but we have no control data to support this hypothesis.

**Figure 2:**
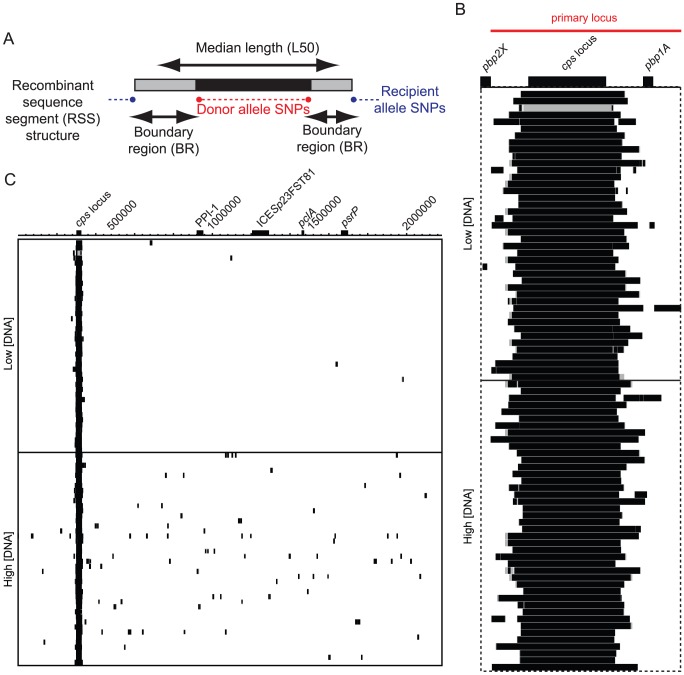
Distribution of recombination events across the genome. (A) Cartoon describing the structure of RSSs as discussed in the text. (B) A detailed view of the RSSs spanning and surrounding the *cps* locus. The annotation of the region is shown at the top, with the *cps* locus and flanking *pbp* genes marked. The red line denotes the extent of the ‘primary locus’ (see text). Underneath, in the panel indicated by the dashed boundary, the RSSs affecting this locus are indicated on the rows by black and grey blocks, as displayed in panel (A). There is a row for each of the 84 transformants, segregated according to the amount of DNA with which they were transformed. (C) Wider view of recombination across the genome. A simplified annotation of the 2,182,009 bp *S. pneumoniae* 23F-R genome is displayed across the top, with the site of the selected recombination (the *cps* locus) labelled along with other major chromosomal loci. The RSSs are displayed as indicated in panel (A), with the strains in the same order as in panel (B).

Recombinant sequence segments (RSSs) were detectable as loci containing donor alleles at polymorphic sites, defining the minimum size of the recombination, bounded by recipient alleles at the flanking polymorphic sites, demarcating the maximum size of the exchange (Figure 2A). The actual length may be estimated as being the median (L50) between these two limits, positioning the flanks half way through each boundary region (BR). As with the separation between any two loci, this length can be expressed either as a distance relative to the donor (L50_D_) or recipient (L50_R_) genome, which differ where there are sequence insertions distinguishing the strains. The selected recombination at the *cps* locus was detected in all transformants. In order to distinguish those events driven by selection from those elsewhere in the chromosome, a ‘primary locus’, encompassing the *cps* region, was defined between the genomic loci 294,383 bp and 340,516 bp in strain 23F-R: all bases between these coordinates had undergone recombination in at least one of the 84 sequenced isolates (Figure 2B).

Furthermore, 107 unselected, ‘secondary’ recombinations were observed outside the primary locus (Figure 2C), with one strain alone having a genome-wide total of 15 RSSs. The mean proportion of the recipient genome found to have undergone recombination was 1.4%, ranging up to a maximum of 2.5%; however, no significant correlation between *in vitro* growth characteristics and the extent of these secondary transfers could be found (Figure S1), although the selected transfer at the *cps* locus did appear to detrimentally affect the growth of all transformants consistently. Secondary recombinations were significantly more common in the strains transformed at a high concentration of DNA (mean of 2.29 secondary events per strain) than at a low concentration (mean of 0.26 secondary events per strain; Wilcoxon test, *p* = 1.39×10^−8^). Hence the effective concentration of DNA available for recombination inside the cell can vary. This implies that recombination events involving separate DNA molecules can occur within the same cell concurrently and independently, as has been previously observed *in vitro* [38], [44] and inferred from the sequences of clinical isolates [45], rather than all arising from the import of a single large molecule of DNA, as has been observed in *S. agalactiae* [46].

### Stochastic resolution of selected recombinations

**Figure 3:**
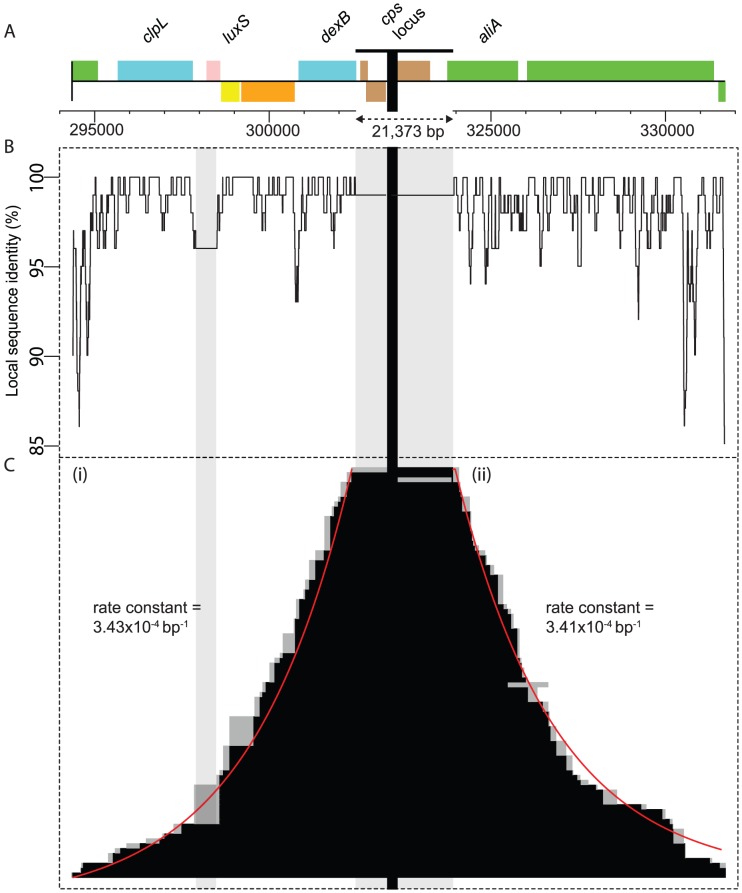
Structure of recombinations within the primary locus. (A) Annotation of two sections immediately flanking the *cps* locus, within the primary locus. The position of the *cps* locus is marked by a horizontal black bar, with a vertical grey shaded band underneath; this locus, across which all recombinations span, is not shown to scale relative to the flanking regions. The extent of an IS element insertion in *S. pneumoniae* 23F-R, not present in *S. pneumoniae* TIGR4**Δ** *cps*, is marked by a second grey vertical band within the 5′ flank. A scale bar, labelled with the genome coordinates and with tick marks every 1 kb, is displayed beneath the annotation. (B) A plot of sequence identity showing the level of similarity between the donor and recipient throughout the locus. The value on the graph represents the proportion of the aligned flanking 100 bp that is identical in both strains for each base in the displayed region. (C) Distribution of recombination sizes. The selected RSSs spanning the *cps* locus from each of the sequenced 84 isolates are displayed as explained in Figure 2A. At the *cps* locus itself, all 84 isolates have the donor allele. On the left side, in (i), the RSSs are ordered by their 5′ boundary, while on the right side, in (ii), they are independently ordered by their 3′ boundary. The positions of these boundaries can be modelled as an exponential decay, which is represented by the red line and displayed rate parameter for each flank.

The high density of SNPs within the primary locus allows a high-resolution view of the boundaries of the selected RSSs that span the *cps* locus (Figure 3). By ordering the selected recombinations by either their upstream or downstream boundaries, the manner in which the primary RSSs end relative to their distance from the selected marker can be observed. This is best modelled as an exponential decay, with similar estimates of the decay constant on both sides: 3.43×10^−4^ bp^−1^ (95% confidence interval, 3.42–3.44×10^−4^) on the left flank, and 3.41×10^−4^ bp^−1^ (95% confidence interval, 3.40–3.42×10^−4^) on the right. The symmetrical nature of the decays on both flanks does not reflect spatial patterns of sequence similarity between donor and recipient, which are poorly correlated to distance from the *cps* locus (Pearson correlation, *R* ^2^ = 2.19×10^−4^). Nor is the decay upstream of the locus strongly affected by an IS element insertion in the recipient sequence. Hence there does not appear to be sufficient local sequence heterogeneity in this region to strongly disrupt the overall patterns of sequence integration.

The exponential declines suggest that the boundaries of RSSs are formed through a Poisson process, occurring with a low, constant probability per base. The assumption of such a mechanism in eukaryotic systems predicted such a distribution should be observed [47]. In the absence of sequence identity affecting the exponential declines, the number of isolates in the recombinant state should halve over each ∼2.4 kb stretch of sequence. On the basis of this rate, of the recombinations that directly affect the *cps* locus, 2.6% will replace *pbp1A* and 3.5% will replace *pbp2X*. However, the only transformant to have one of these genes (*pbp1A*) completely replaced was still able to grow, albeit at a greatly reduced rate, on ampicillin. Less than 0.1% of selected recombinations encompassing the *cps* locus would be expected to replace both *pbp2X* and *pbp1A* in their entirety, appearing to explain the lack of a readily observable inhibition of *cps* transfer by ampicillin selection.

### Non-contiguous recombinations from a single donor molecule

However, this is an oversimplification, as recombinant sequences can be found at high density around the selected recombination, where they are likely to affect the *pbp1A* and *pbp2X* genes. Thirty-six further RSSs occur in the primary locus but do not span the *cps* gene cluster (Figure 2B). This concentration of unselected primary recombinations suggests that they are associated with that spanning the *cps* locus, forming a non-contiguous recombination (NCR) as has previously been observed on a smaller scale in the generation of penicillin-resistant *pbp* alleles *in vitro* [34]. This may represent a consequence of the recipient genome's properties, with restricted regions of the chromosome capable of undergoing recombination at high rates perhaps due to local chromatin arrangements or supercoiling properties. Under such circumstances, the flanking RSSs may arise from different donor molecules to the selected RSS. Alternatively, it may result from a single donor molecule being integrated in a non-contiguous manner, in which case these flanking events will have arisen from the same imported DNA as the acquired selected marker. The latter hypothesis is supported by the observation that strains transformed with the lower concentration of DNA actually have a larger mean number of primary RSSs (1.52 per strain) than those exposed to the higher concentration (1.33 per strain), although this difference is not significant (Wilcoxon test, *p* = 0.33). This higher density of recombinations in the cells exposed to a lower concentration of donor DNA is the opposite of what would be expected if donor molecules could independently contribute to the genetic exchange within this locus, indicating that a single piece of DNA acts as the origin for multiple RSSs. While none of the selected RSSs affected the *pbp* genes to a large extent, two and four unselected primary RSSs overlapped with *pbp2X* and *pbp1A*, respectively, demonstrating how mosaicism complicates the issue of linkage between loci and increases the likelihood of capsule switches being associated with changes in penicillin resistance.

**Figure 4:**
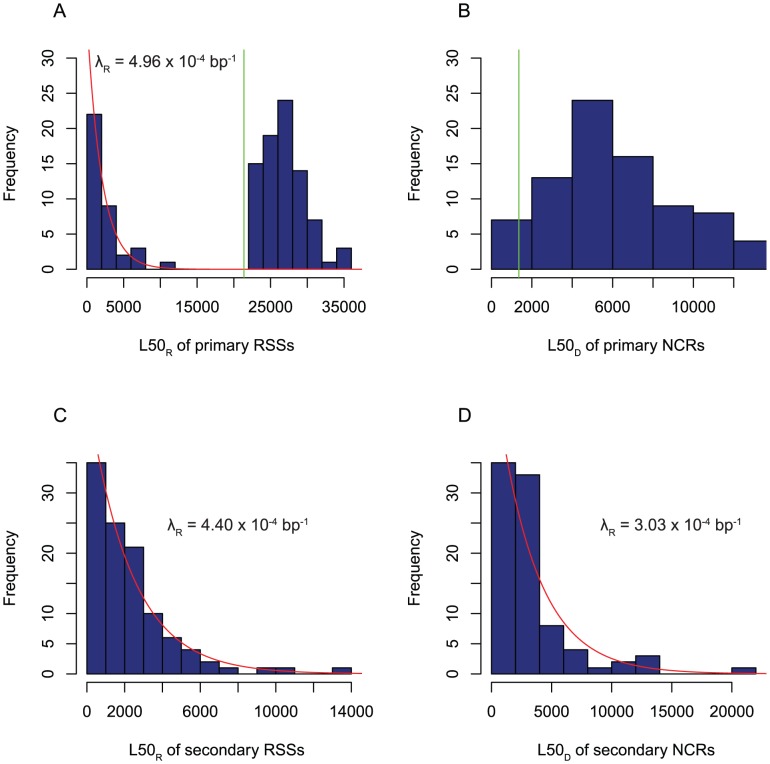
Distribution of recombination sizes. (A) Histogram showing the distribution of RSS sizes as L50_R_ lengths. The vertical green line shows the length of the *cps* locus in the recipient's genome (21,373 bp). The recombinations longer than this are the selected events importing the kanamycin resistance marker. The smaller, unselected recombinations are modelled as an exponential distribution (red line), with the calculated rate parameter displayed. (B) Histogram showing the distribution of donor molecule lengths participating in primary recombinations, as estimated using the L50_D_ lengths of the NCR boundaries. The vertical green line shows the length of the kanamycin resistance locus in the donor DNA (1,354 bp). (C) Histogram showing the distribution of secondary RSS sizes as L50_R_ lengths. These are modelled as an exponential distribution, indicated by the red line and displayed rate parameter. (D) Histogram showing the distribution of secondary NCR L50_D_ lengths, thereby estimating the sizes of donor molecules participating in secondary recombinations. These are also modelled as an exponential distribution, indicated by the red line and annotated rate parameter.

A histogram of the L50_R_ values for recombinations within the primary locus reveals the selected and unselected RSSs have very different size distributions (Figure 4A). The unselected RSSs are typically less than 5 kb in length, and follow an approximately exponential distribution with a rate constant, λ_R_, of 4.96×10^−4^ bp^−1^ (95% confidence interval 3.60–7.23×10^−4^ bp^−1^). Those spanning the *cps* locus are longer (and therefore estimate λ_R_ as being smaller), due to the necessity of spanning the 21,373 bp gene cluster, and follow a different size distribution, as the probability of transmitting the selectable marker to the recipient rises as the length of the recombination increases [47]. The size distribution of the imported strand, which can be calculated on the basis that all primary RSSs originate from the same DNA molecule, is similar (Figure 4B). The median L50_D_ of these values was found to be 5.9 kb, comparable to the median imported strand length of ∼6.6 kb [9] despite the size constraint of importing the kanamycin resistance gene.

### Sequence identity does not affect positioning of secondary recombinations

The size distribution of the secondary RSSs was also found to resemble an exponential distribution (Figure 4C); the larger number of datapoints in this set allowed this assessment to be confirmed quantitatively (Figure S2). The implied λ_R_ of 4.40×10^−4^ bp^−1^ (95% confidence interval 3.71–5.38×10^−4^ bp^−1^) is similar to that deduced from the unselected primary recombinations and reflects the mean length of these RSSs, 2.27 kb. To test whether they also exhibited the same mosaicism as observed in the primary locus, a bootstrapping analysis (see Methods) was used to compare distances between RSSs within the same isolate against the distribution of distances between RSSs in different isolates. This test was designed to find RSSs in an isolate that were significantly closer together than would be expected by chance, indicating that they are likely to have originated from the same important molecule of DNA, and link them together into NCRs. The outcome grouped the 107 secondary RSSs into 87 NCRs, which contained up to three segments of imported sequence; like the component RSSs, NCRs were significantly more frequent in strains transformed at a high concentration of DNA (Wilcoxon test, *p* = 5.68×10^−9^). Those secondary NCRs composed of more than one RSS were estimated to be composed of approximately half donor sequence (mean proportion of 55%), with the remainder unmodified recipient sequence. However, the L50_D_ lengths of the NCRs did not appear to fit an exponential distribution as well as the RSS lengths (Figure 4D). If they had, it might have suggested each NCR was formed through a Poisson process, with the component RSSs produced through post-processing of this larger structure; as it is, the data indicate the RSSs are formed through a Poisson process, with the composite NCRs more irregular in their form.

**Figure 5:**
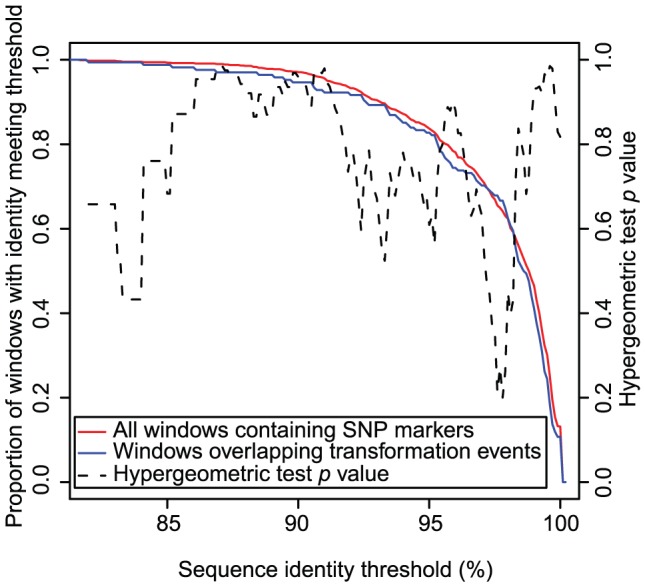
Distribution of secondary recombinations with respect to sequence divergence between the donor and recipient strains. The red line represents the proportion of non-overlapping windows of the recipient sequence, containing at least one marker SNP, that have a mean SNP sequence identity of, or greater than, the threshold marked on the x axis. The blue line represents the same statistic for the subset of windows that overlap with at least one detected secondary RSS. The two lines are very similar, suggesting that recombinations are not concentrated in a subset of windows that have a relatively high level of sequence identity between the donor and recipient. A hypergeometric test for enrichment of recombinations in windows with high levels of sequence similarity was performed at 0.1% intervals; the calculated *p* values are displayed as a black dashed line relative to the y axis on the right side, confirming the lack of a significant relationship.

Being distributed around the chromosome, the secondary RSSs provided an opportunity to test whether sequence similarity between the donor and recipient affects the positioning of genetic exchanges in this system. Dividing the entire recipient genome into 1,000 equally-sized non-overlapping windows produced 890 outside the primary locus that were found to contain at least one marker SNP, and therefore had the potential to contain a detectable recombination. Comparing the distribution of mean SNP sequence identities (calculated using 50 bp of aligned sequence on each side) of all such windows against the subset of 168 that were found to overlap with secondary RSSs (Figure 5) provided no evidence for the enrichment of imported sequence in regions of the genome with greater sequence identity between donor and recipient. A ‘walking hypergeometric test’ performed at different sequence identity thresholds confirmed the absence of a statistically significant relationship, which concurred with a more detailed sliding window analysis (see Text S1).

The previously reported effects of sequence identity may reflect the necessity for MEPS extending beyond a threshold length, estimated at a total across both ends of 27 bp for *S. pneumoniae* [27]. The results of this experiment are almost entirely consistent with this constraint, except for a single RSS that transfers one SNP and has BRs totalling 26 bp in length. However, the sliding window analysis failed to find a significant enrichment of RSSs with BRs that were longer than expected by chance alone (see Text S1). This is likely to reflect the low density of markers across the recipient genome relative to the MEPS threshold, suggesting that the frequency of polymorphisms across the pneumococcal genome in this experiment is not high enough to strongly inhibit most transformation events.

One other possible characteristic of the genome that might influence the positioning of recombinations is the presence of small interspersed repeats. However, comparing the density of BOX, RUP and SPRITE sequences [48] within and outside secondary RSSs failed to uncover any significant associations (Table 1).

**Table 1:** The distribution of insertions, deletions and small interspersed repeats relative to RSSs.

Sequence Feature	Inside Secondary RSSs	Inside Secondary BRs	Outside Recombinations	*P* value, density inside RSSs	*P* value, density inside BRs
Bases	192,122	97,593	1,626,983	-	-
BOX	11	8	94	1.00	0.29
RUP	10	4	79	0.86	1.00
SPRITE	1	0	25	0.52	0.40
Insertions	41	20	466	0.071	0.17
Deletions	40	11	391	0.43	0.0091

The total numbers of core genome positions (*i.e.* the length excluding sequence insertions in either the donor or recipient strain) in the segments of imported donor sequence and BRs of secondary RSSs were separately calculated, as was the number of core genome positions that were not found to be affected by RSSs in any strain. These were compared with the number of repeats (counting only instances of these elements shared between donor and recipient), insertions and deletions relative to the recipient that were categorised in the same way. Where RSSs in different strains overlapped, bases and features were counted multiple times.

### Polymorphisms affecting the positioning of recombinations

**Figure 6:**
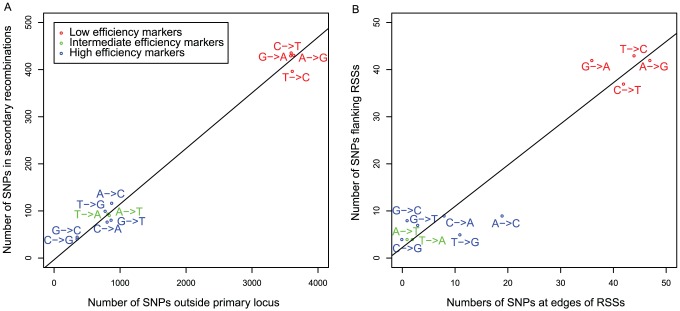
Frequencies of different base substitutions within secondary recombinations. (A) Scatterplot showing the incidence of different substitutions outside the primary locus relative to their frequency in secondary recombinations. Points are coloured according to the efficiency with which they are transferred through transformation when mismatch repair is active (see key). This shows that SNPs repaired effectively by mismatch repair are the most common, and are represented in secondary recombinations at the same level as those SNPs repaired less effectively. (B) Scatterplot showing the frequency of different substitutions when imported at the extreme ends of RSSs (x axis) relative to their frequency as the nearest flanking marker SNP, which is not converted to the donor allele (y axis); this compares the types of substitution found at the two polymorphic sites that define BRs. This fails to provide any evidence that localised mismatch repair processes may affect the positioning of transformation event boundaries or be the cause of the mosaic structure of recombinations, as SNPs repaired more efficiently by mismatch repair are approximately equally represented within, and surrounding, secondary RSSs.

Past studies focussing on individual loci have found that MMR strongly influences the transfer of small number of polymorphisms [12], [14], [30]. However, as just a few hundred SNPs appear to be sufficient to overwhelm the pneumococcal MMR system [26], it is not clear whether this this form of repair is likely to have extensively impacted on recombinations in this system. To test this, the number of each type of SNP outside the primary locus was compared with the frequencies of these mutations in the secondary recombinations, resulting in the observation of a tight correlation (Pearson correlation *R^2^* = 0.99, *p* = 2.83×10^−12^; Figure 6). Furthermore, the frequency of each SNP on the outermost position of each RSS is proportional to its prevalence in the nearest flanking unchanged position (the two positions defining BRs; Pearson correlation *R^2^* = 0.92, *p* = 7.39×10^−7^). Hence there is no evidence that the low efficiency markers, acted on most effectively by MMR, lead to entire recombinations being lost at a higher frequency, or that they trigger localised repair, which might have been a mechanism for the formation of NCRs. These graphs do show, however, that the most frequent mutations found distinguishing the donor and recipient strains, independent of the observed transformations, are the transversions, which are targeted most efficiently by mismatch repair. Contingent upon the majority of these polymorphisms having arisen through single step mutations, this supports the hypothesis that MMR has evolved to repair the most frequent mutations most efficiently [49].

By contrast, insertions and deletions (indels) appear to have a stronger effect on the positioning of RSSs. Within secondary RSSs, insertions (relative to the recipient) are underrepresented, although not to a statistically significant extent (Table 1). However, these insertions within RSSs are significantly smaller than the rest of the identified insertions outside the primary locus that are not transferred in this experiment (Wilcoxon test, *p* = 0.015). However, deletions relative to the recipient within secondary RSSs show no such significant difference in sizes compared to those outside transformed sequences (Wilcoxon test, *p* = 0.86). This is not likely to be an artefact of the overall distribution of deletion and insertion sizes, which do not differ significantly (Wilcoxon test, *p* = 0.32). Conversely, deletions were found to be significantly underrepresented in BRs (Table 1), and both insertions and deletions in BRs were significantly longer than those outside recombinations (Wilcoxon tests, *p* = 0.0010 for insertions and *p* = 0.027 for deletions). Manual inspection of the mapped sequence read data found that the majority of these indels remained of the recipient allele and therefore had not been cotransfered with the neighbouring RSS. Combining these results concerning BRs suggests that small differences in length between the invading strand and recipient genome may strongly inhibit the formation of recombination edges, implying that indels can only be maintained in a heteroduplex if stabilised on either side by loci of matching lengths in the donor and recipient genotypes.

### Absence of influence of mismatch repair

**Figure 7:**
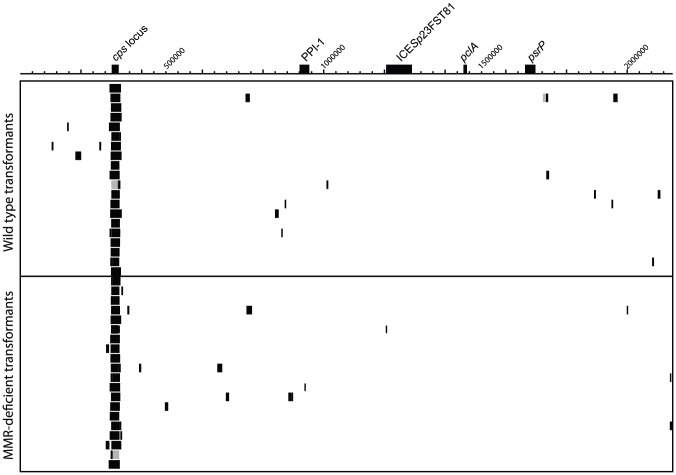
Distribution of recombination events across the genome when testing the effect of MMR, displayed as described in Figure 2C. The transformants are grouped according to their genotype, either wild type or mismatch repair defective. Both were transformed with 500 ng mL^−1^ donor DNA.

In order to experimentally verify the apparent lack of a role for MMR in moderating the exchange of sequence in this *in vitro* system, and ascertain whether it may be involved in any localised repair mechanism responsible for the formation of NCRs, a transformation experiment was performed using the same donor DNA, at 500 ng mL^−1^, and selection on kanamycin alone, with an MMR-deficient recipient (*S. pneumoniae* 23F-RΔ*hexB*) alongside the wild type strain. Twenty isolates of each genotype were analysed as described for the first experiment (Figure 7), resulting in the identification of 1,463 polymorphic sites. Of these, 92 did not correspond to marker SNPs; the majority of these were found in the MMR-deficient strains, suggesting the loss of the repair functionality resulted in an increased rate of point mutation accumulation during culturing.

Identification of recombinant sequence demonstrated that there was no significant difference between the number of primary RSSs in the wild type (mean of 1.50 per strain) and MMR-deficient (mean of 1.65 per strain; Wilcoxon test, *p* = 0.90) strains. This indicates that MMR does not appear to have a role in generating the mosaic structure of recombinations. This is congruent with both the previous observations that the low efficiency markers are found at equal frequencies on either side of recombination boundaries, indicating these polymorphisms are not targeted by localised MMR, and that the distributions of secondary RSS lengths fit an exponential distribution more closely than those of the secondary NCR lengths, implying the RSSs form independently rather than emerging through partial repair of NCRs. Furthermore, there was no difference in the mean number of secondary RSSs between the wild type (mean of 0.85 per strain) and MMR-deficient (mean of 1.00 per strain; Wilcoxon test, *p* = 0.94) isolates or in the mean lengths of these secondary recombinations (Wilcoxon test, *p* = 0.71). This confirms that under these conditions, with the genotypes used in this experiment, MMR does not appear to be moderating transfer of sequence between donor and recipient.

## Discussion

Many previous studies of transformation have necessarily focused on the rates of transfer of a small number of selectable mutations. The advantage of high-throughput genome sequencing, coupled with a controlled *in vitro* system, is the ability to characterise the full extent of multiple recombinations occurring throughout the chromosome. Using these data, it can be observed that multiple RSSs can be generated from a single donor molecule of DNA, thereby forming NCRs. Furthermore, multiple NCRs, each from a different donor molecule, can be generated during a single period of competence. Recent work on a smaller sample of *Haemophilus influenzae* transformant genomes [50] concurs with this conclusion, as well as the lack of effect of sequence diversity on the distribution of recombinations, although unfortunately the study could not evaluate these points statistically due to sample size constraints. However, the recombination lengths in the *H. influenzae* transformants appear to follow a different distribution to those described here in *S. pneumoniae*.

The exponential length distribution apparent from this work suggests the boundary of the event is determined by random termination with a fixed per-base probability of λ_R_. The relatively weak observed linkage between the *cps* locus and the *pbp1A* and *pbp2X* genes is a consequence of this size constraint. It is not clear what mechanism is responsible for this Poisson process: it seems likely to be the resolution of the heteroduplex intermediate, but could alternatively represent a process such as cleavage of the imported strand. The consequent exponential distribution has potentially interesting implications for bacterial evolution, as it suggests that transformation is optimised for transferring short lengths of similar sequence, rather than replacing large genetic features, such as operons, with homologous sequence in a single event. The calculated value for λ_R_ suggests over a third of RSSs will be less than 1 kb, the approximate size of a typical bacterial gene. This is likely to have been crucial in facilitating the generation of mosaic penicillin-resistant *pbp* genes through incorporating fragments of penicillin-sensitive *S. mitis* and *S. oralis* orthologues into the penicillin-sensitive *S. pneumoniae* versions [51], [52]. Longer imports would have simply exchanged one sensitive allele for another. It will be interesting to observe whether proteinaceous antigens in *S. pneumoniae* display a similar mosaicism as a result of transformation.

Does this mean large events, such as serotype switching, will occur only rarely in the population? This depends, to a great extent, on the important consideration of whether the λ_R_ per base probability applies to the nucleotides of the incoming strand, the recipient genome, both, or only those bases that are paired between the incoming and resident DNA (of course, there may be other effects of indels on heteroduplex stability that are independent of λ_R_). This will largely determine how recombination behaves with regard to indels. If λ_R_ applies to the incoming DNA, then recombinations would presumably abort when terminated within an insertion in the donor relative to the recipient, due to the absence of a homologous locus in the recipient chromosome. This would lead to insertions being underrepresented in RSSs at a level related to their length. Conversely, if λ_R_ applies to the host DNA bases, then deletions in the donor relative to the recipient will be underrepresented in an analogous fashion.

However, if λ_R_ applies either to only bases that pair in the formation of recombinant segments, or equally to insertions in both genotypes, then the Poisson process would not cause any asymmetry between insertions and deletions in the donor. Yet there would be an important difference at sites where one gene cassette replaces another (*e.g.* at the *cps* locus), as in the former case the presence of the indels is potentially negligible, whilst in the latter case the lengths of the incoming and resident alleles would both contribute to inhibiting the transfer. However, it would be expected that an overall bias against insertions in the donor, relative to deletions, would be observed in both cases, as the physical size of the insert on the donor DNA makes it susceptible to cleavage upon import, whereas deletions cannot be degraded upon entry in the same way [53].

Previous work in *S. pneumoniae* has found, in reciprocal crosses, that length polymorphisms are imported more efficiently as deletions rather than as insertions [24], [25], [54], although a more recent study did not replicate this result [53]. This is congruent with any of the outlined scenarios, except that where the Poisson process involves bases of insertions in the recipient but not the donor. In this context, it may be informative to note that the longest predicted homologous recombination found in the sequencing of over 200 PMEN1 strains was an event that deleted the ∼38 kb genomic island encoding the *psrP* antigen [35], suggesting its extreme length may be explained through the bases of the insertion in the recipient not being taken into account by the Poisson process that determines the length of RSSs.

The data described in this work found that RSSs were less likely to contain long insertions than deletions, in agreement with the aforementioned studies that suggest a bias against the acquisition of insertions. This suggests that the net effect of imported DNA cleavage, heteroduplex formation and resolution of the recombination is a tendency towards importing shorter alleles, rather than gain of longer sequences. If correct, when combined with the exponential distribution of recombination sizes, this would mean a ‘molecular drive’ would exist that would lead to the fixation of the shortest allele at a locus in the absence of selection for the sequences being deleted. This may provide an explanation for the observed, but not fully explained, deletional bias among prokaryotes [55], and suggests that the maintenance of multiple alleles that are polymorphic in length, as observed with the different capsule biosynthesis gene clusters at the *cps* locus, should be rare in a transformable bacterial population. However, exactly how infrequent they are expected to be, in the absence of selection, cannot be determined until more precise details of the transformation mechanism are understood.

**Figure 8:**
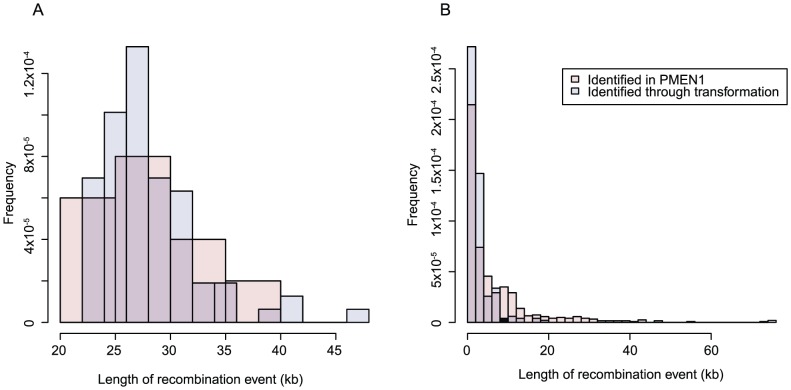
Histogram comparing frequencies of detected recombination sizes in a collection of PMEN1 isolates and *in vitro* transformants. The algorithm used to detect sequence imports in a sample of PMEN1 genomes was individually applied to each transformant sequence from the first experiment described in this work. In both graphs, the blue bars show the output of the algorithm applied to sequences from this experiment, whereas the red bars represent the data from the PMEN1 strains. (A) Comparison of the ten serotype switching recombinations characterised in PMEN1 with the transformation events spanning the *cps* locus selected *in vitro*. (B) Comparison of the unselected secondary transformation events characterised *in vitro* with the 615 homologous recombinations (*i.e.* excluding events overlapping prophage or conjugative elements) identified in the PMEN1 isolates.

The lengths of the recombinations changing capsule type in the PMEN1 clinical isolates can be compared with those selected in this work through applying the algorithm used to detect sequence imports in the PMEN1 strains [35] to the *in vitro* transformants. In these comparable datasets, the distribution of *cps*-spanning recombination sizes is very similar in both (Figure 8A). Elsewhere in the genome, the distributions of recombination sizes are alike (Figure 8B), but the set detected in the PMEN1 population has fewer short recombinations. This is likely to be due to a reduced power to differentiate such events, which import few SNPs, from the background of point mutations in the clinical isolates, as compared to the near-complete absence of variation outside of transformation events from the isolates in this study. Hence the exponential decline is less clear from the data derived from clinical isolates, and the rate parameter λ_R_ is estimated as being artefactually low (1.58×10^−4^ bp^−1^; 95% confidence intervals 1.47–1.72×10^−4^ bp^−1^).

Overall, recombinations were found to affect almost three-quarters of the reference genome length in the PMEN1 sample, which is congruent with the apparently random positioning of RSSs seen in this experiment. This would appear to be a consequence of the divergence between donor and recipient (a mean density of one per 96 bp of sequence aligned between the donor and recipient). The import of sizeable DNA fragments from a genotype of such diversity into the studied recipient may be expected to include sufficient SNPs to overwhelm the MMR system [26], while not containing a high enough density of polymorphisms to commonly restrict the resolution of a heteroduplex [27]. Hence both these potentially restrictive processes seem likely to be relatively uninfluential at this level of sequence divergence, with no evidence of either observed in this experimental setup.

Some factors need be considered before applying these results to all pneumococcal transformations. For instance, it may be that in the absence of selection for such a large recombination at the *cps* locus, MMR may be more of a barrier to sequence transfer at low concentrations of DNA, when the cell imports less genetic material and therefore fewer polymorphisms. The disruptive effects of the selected transformation may also have been accentuated by the large different in length between the incumbent *cps* locus and small selected marker. Additionally, it is possible the experimental conditions used may not have induced expression of the MMR system in the recipient strain; it has been observed that pneumococci vary in the rate at which they import sequence [56], which could potentially reflect differences in the overall activity or regulation of MMR in different strains. The use of other genotypes may have lead to stronger signals of MMR being identified in this experiment.

The level of diversity found distinguishing the donor and recipient will also affect the pattern of observed transformation events, although the mean divergence between the strains in this experiment is typical of that observed when comparing pneumococci [41]. Aligning all available complete pneumococcal genomes to the recipient reveals a minimum and maximum SNP density of one SNP per 150 bp (*S. pneumoniae* JJA) and one SNP per 81 bp (*S. pneumoniae* Hungary 19A-6), respectively. By contrast, a comparison with *S. mitis* B6 [57] with the recipient sequence reveals a SNP density of one per 23 bp, enough to frequently interfere with the formation of a recombination edge, according to the MEPS model. Hence while the constraints of MMR and sequence diversity will impact on within-clone and between species transfers respectively, the positioning and selective advantage of accessory genome loci are likely to have the greatest mechanistic impact on homologous recombination between lineages of the pneumococcal species.

## Methods

### Statistical analyses

All statistical analyses were performed using R. The “fitdistrplus” package was used to find distributions to experimental data and establish 95% confidence intervals through bootstrapping analysis. The “grofit” package was used to extract statistics from growth curves. As data analysed in this study tended not to be normally distributed, non-parametric statistical tests were used throughout.

### Generation and alignment of reference genome sequences

The TIGR4Δ*cps* strain was previously created through replacing the *cps* locus of *S. pneumoniae* TIGR4 with a kanamycin resistance marker [40]. A draft genome assembly for strain TIGR4Δ*cps* was generated by using the sequence of the disrupted capsule biosynthesis locus reported following the construction of this strain (EMBL accession code AF160759) to replace the serotype 4 capsule locus of the TIGR4 genome (multilocus sequence type 205; EMBL accession code AE005672) [58]. This hybrid sequence served as an initial draft of the TIGR4Δ*cps* genome, which was then corrected with ICORN [59] using Illumina sequence data generated from the donor DNA used in the transformation experiments. This resulted in the correction of 56 base substitutions and 20 single base indels. The sequence of *S. pneumoniae* 23F-R was derived by correcting the reference sequence of *S. pneumoniae* ATCC700669 (multilocus sequence type 81; EMBL accession code FM211187) [39] with ICORN using resequencing data [35]; this revealed the presence of eight substitutions, one single base indel and the loss of prophage ΦMM1-2008. The finalized genomes were aligned using MUGSY [60] resulting in 21,541 marker polymorphisms being identified. To avoid the false positive identification of transformation events, polymorphisms within the four rRNA operons, the hypervariable *hsdS* locus [58] and highly repetitive *psrP* gene [39] were excluded from all analyses. This left 20,773 marker SNPs for use in detecting RSSs. In order to remove any of these sites that would be liable to lead to the false positive inference of recombinations when analysing Illumina sequence reads, the data generated from the donor DNA (used to correct the sequence of strain TIGR4Δ*cps*) were mapped to the corrected sequence of the recipient, strain 23F-R. Using the criteria described in Harris *et al.* [42], 17,534 SNPs could be identified, all of which, except 385 false positives, corresponded to polymorphisms identified through the whole genome alignment. The false positive positions were excluded from subsequent analyses to avoid erroneous inference of transformation events. An evaluation of the SNP calling parameters was performed using these data; this found that the identification of polymorphisms did not alter greatly except when using the most extreme choices of parameter selection (Figure S3).

### Transformation experiments

Agarose gel electrophoresis was used to check that donor DNA used in these experiments was not heavily degraded. Overnight cultures of *S. pneumoniae* 23F-R were diluted 1∶100 into BHI (Oxoid) and grown statically at 37°C to an optical density of OD_600_ between 0.20–0.25. A 1 mL sample of the culture was then added to 10 ng competence stimulating peptide 2 (CSP-2; Sigma), 5 µl 500 mM calcium chloride and 5 µl of an aqueous solution of donor DNA, at a concentration of either 1 µg mL^−1^ or 100 µg mL^−1^. These reactions were incubated at 37°C for 2 h, and then three 50 µL volumes each spread onto 5% horse blood agar plates supplemented with 200 g L^−1^ kanamycin (Gibco), and three further 50 µL volumes each spread onto 5% horse blood agar plates supplemented with 200 g L^−1^ kanamycin and 100 mg L^−1^ ampicillin (Sigma). Colonies were counted and picked after 16 h incubation at 37°C.

### Sequencing of isolates

The first experiment involved six transformations at the lower concentration of DNA and three transformations at the higher concentration, along with a negative control containing no CSP-2 that generated no kanamycin-resistant colonies. DNA was prepared from randomly-picked colonies, and sequenced as multiplexed libraries of 12 tags on an Illumina Genome Analyzer II as described in Croucher *et al.* [35] to give 76 nt paired end reads.

The second experiment involved three transformations of the wild type and mutant strains as described above. DNA was prepared using an Xtractor Gene system (Qiagen) and sequenced as multiplexed libraries of 24 tags on an Illumina HiSeq, according to manufacturer's instructions, to give 76 nt paired end reads. Four datasets each from the wild type and mutant groups were discarded as they showed potential signs of contamination.

### *In vitro* characterisation of isolates

For each of the 84 sequenced transformed strains from the first experiment, along with the donor and recipient strains, an overnight culture growing in 10 mL BHI (Oxoid) was diluted 1∶100 into 5 mL sterile BHI. A 300 µL sample of each freshly inoculated culture was then immediately transferred into a 96 well plate, and growth was followed for 12 h at 37°C by measuring the OD_600_ of each well using a FLUOstar Omega microplate reader. Three independent biological replicates of this experiment were performed.

### Construction of *S. pneumoniae* 23F-RΔ*hexB*

Regions around 500 bp in length on either side of *hexB*, both including *Bmg*BI cut sites encoded by BOX elements, were amplified by PCR (all primer sequences are listed in Table S3) and cloned into pGEM-T Easy, according to manufacturer's instructions, to give plasmids pLeft and pRight. The *ermB* erythromycin resistance gene was then amplified from strain *S. pneumoniae* 11930 [35] using the primers ermBL and ermBR (each with an *Eco*RV cut site on the 5′ end), and cloned into pGEM-T Easy as described above to give plasmid pErm. All three plasmids were then transformed into *E. coli* TOP10 cells (Invitrogen) through electroporation with a 2.5 kV pulse, followed by blue-white selection on 100 µg mL^−1^ ampicillin. The plasmid carrying the *ermB* gene was then extracted using a QIAprep Spin Miniprep Kit (Qiagen) and digested using *Eco*RV (New England Biolabs), releasing the insert with blunt ends. This fragment was purified through agarose gel electrophoresis using a QIAquick Gel Extraction kit (Qiagen). Plasmid pRight was similarly extracted, then digested with *Bmg*BI (New England Biolabs), which also cuts to give blunt ends. The *ermB* fragment was cloned into this blunt cut site using T4 ligase (Promega) overnight at 4°C according to manufacturer's instructions. A 1 µL sample of this reaction was then used to transform electrocompetent *E. coli* TOP10 cells as described above. A 50 µL sample of this culture was then spread on LB agar plates supplemented with 100 µg mL^−1^ erythromycin. The plasmid pErmR was isolated from a colony, and its composite insert and the adjacent multiple cloning site (MCS) amplified using primers ermBL (which has an *Eco*RV cut site on the 5′ end) and T7. This amplicon was purified through agarose gel electrophoresis as described above and digested with *Eco*RV and *Nco*I (New England Biolabs), the latter of which cuts within the MCS of pGEM-T Easy. This was ligated into pLeft following its digestion with *Bmg*BI and *Nco*I as described above. This ligation reaction was used to transform *E. coli* TOP10 cells as described above, with strains carrying the plasmids again selected on LB agar supplemented with 100 µg mL^−1^ erythromycin. This gave pErmLR, which was used to transform *S. pneumoniae* 23F-R. Pneumococcal transformants with disrupted *hexB* genes were selected on 5% blood agar plates supplemented with 0.1 µg mL^−1^ erythromycin. One of these colonies was picked and the insert checked through PCR amplification with primers hexBL and hexBR and capillary sequencing. Further confirmation was provided through *de novo* assembly of Illumina sequence reads generated from 23F-RΔ*hexB* transformant DNA. This scheme is summarised in Figure S4.

### Identification of recombinant sequences

Illumina data from transformants were independently mapped to the corrected genomes of 23F-R and TIGR4Δ*cps* using SMALT [61]. The *de novo* identification of SNPs, to check for mutations not derived from imports of the donor sequence, was performed by identifying polymorphisms relative to the recipient genome as described in Harris *et al.* [42]. All strains had a mean sequence read coverage of at least 20 fold, with the mean coverage across the dataset of 123 fold, for strains sequenced using the Genome Analyzer II, and 506 fold, for strains sequenced using the HiSeq.

To identify RSSs, only marker SNP sites were analysed. The alignment produced by SMALT was processed using samtools [62] and vcftools [63] to produce Variant Call Format (VCF) outputs for each strain. Within these VCFs, all homozygous marker sites with a base quality greater than 50 were used to identify sequence characteristic of the donor or recipient in the transformation experiment. Recombinations were initially defined as regions containing donor alleles at polymorphic sites with no intervening recipient alleles. To exclude false positives, putative recombinations were rejected unless there was evidence of the transfer of at least one of the supporting marker sites from the reciprocal mapping to the donor sequence, similar to the method described by Mell *et al.* [50]. The ambiguous boundary regions around each recombination extended between the outermost donor allele SNPs identified in the transformant and the nearest flanking sites that were found to have recipient allele SNPs. All sequenced transformants were found to have a unique pattern of transformation events, and would therefore appear to represent independent samples of the variation produced the transformation experiments.

### Amalgamation of recombinant sequences into non-contiguous recombinations

Many secondary RSSs in the same strain were positioned very close to one another, suggesting they may have arisen from the same molecule of donor DNA. A bootstrapping approach was used to test whether such apparent spatial associations were significant. To produce a population of test values, the shortest distance (in terms of the donor genome) between all secondary RSSs from all strains in the first experiment was calculated. Then, for each strain with more than one secondary RSS, the shortest distance between the two closest secondary RSSs in the strain was calculated, in terms of the donor sequence, to give the test value (*d*_test_). A bootstrapped sample, equal in size to the test population, was then obtained from the test population, and this distribution used to test the hypothesis that *d*_test_ was significantly shorter than expected under the null hypothesis (*H*_0_) of recombinant sequences being positioned at random relative to one another. Multiple testing was accounted for by using a Holm-Bonferroni correction to alter the one-tailed threshold *p* value of 0.05 according to the number of secondary RSSs in the strain. One hundred such bootstrap tests were performed for each for each *d*_test_, with distances rejecting *H*_0_ on 95 or more of these trials considered to be significantly close to one another and therefore likely to have arisen from the mosaic incorporation of the same molecule of donor DNA. If the first *d*_test_ in a strain was accepted, then the second smallest distance was tested with the appropriate corrected *p* value threshold; this process continued until *d*_test_ was accepted under *H*_0_. Where secondary RSSs were significantly closer than expected under *H*_0_, they were grouped into NCRs.

## Supporting Information

Figure S1 *In vitro* growth characteristics of transformed isolates. The displayed data points, represented as error bars encompassing one standard error of the mean above and below the mean value, summarise three independent biological replicates of growing the transformed isolates in Brain-Hearth infusion broth. Maximum growth density and rate measurements from each replicate were separately scaled relative to the mean value across all strains in the experiment to account for any systematic differences between the replicates. Data for transformants are shown in black; the corresponding points for the donor strain (TIGR4Δ*cps*) are in blue and for the recipient strain (23F-R) in red. The maximum density to which the isolates grew is shown relative to the total number of SNPs transferred through transformation in (A), and relative to the number of non-synonymous SNPs transferred through transformation in (B). In both cases, the datapoint for the donor strain is shown at the extreme right of the x axis, although 20,773 total marker SNPs, and 5,246 nonsynonymous SNPs, actually distinguish it from the recipient. The green line shows the maximum growth density modelled as a linear function of the x axis in each case. In both cases, no significant correlation was found (Pearson correlations *R* ^2^ = 7.14×10^−4^ and 3.50×10^−4^, *p* values = 0.81 and 0.87, for all marker SNPs and non-synonymous marker SNPs respectively). All transformants have a lower peak growth value than both the donor and recipient strains; this appears to be the consequence of the selected recombination, as it is a trait common to all isolates. This indicates there may be an aspect of the recipient genotype that interacts detrimentally with the acapsular phenotype or the expression of kanamycin resistance. The maximum rate at which the isolates were found to grow in the same experiments is shown relative to the total number of SNPs transferred through transformation in (C), and relative to the number of non-synonymous SNPs transferred through transformation in (D). Again, the donor datapoint is shown at the far end of the x axis, and the green lines display the calculated linear relationship between the maximum growth rate and x axis values. Once more, no significant correlation was found in either case.
(PDF)

Figure S2 Distribution of transformation event lengths. This Cullen-Frey plot describes the skewness and kurtosis of the L50_R_ measurements of secondary RSSs. The bold red point indicates the properties of the overall dataset; the pale pink points represent 1,000 bootstrapped replicates from the same dataset. The bootstrapped data points run along the line that suggests the data are described by a gamma distribution; their clustering suggests an exponential distribution, a specific case of the gamma distribution, is appropriate in this case.
(PDF)

Figure S3 Evaluation of SNP identification parameters. These graphs plot the change in the false positive rate (red) and false negative rate (blue) with changes in the value of mapping parameters. The false positive and negative rates were assessed by comparing the set of polymorphisms identified by mapping the Illumina data from the sequencing of the donor DNA against the reference set identified through the whole genome alignment. On each plot, the parameter value used in previous publications [35], [42] is indicated by a vertical dashed line. The parameters evaluated are (A) mapping quality threshold for including a read in the mapping analysis, (B) base quality threshold for use of a read base in SNP calling, (C) minimum number of mapped sequence read bases needed to call a base, and (D) the minimum proportion of the mapped sequence reads that must support a base call at an individual reference nucleotide for the base to be identified. These data show that altering these parameters generally has relatively little impact on the sensitivity and specificity of SNP identification. It should be noted that the false positive and negative rates calculated in this evaluation correspond to that which may be expected when mapping Illumina reads from one pneumococcal isolate onto the reference chromosome from a divergent genotype. They do not reflect the rates expected when identifying SNPs in the transformant sequences, which are much more closely related to the recipient 23-F strain used as the reference genome.
(PDF)

Figure S4 Construction of *S. pneumoniae* 23F-RΔ*hexB*. (A) (i) Upstream and downstream regions flanking *hexB*, both containing *Bmg*BI cut sites within BOX elements (indicated by red boxes), were amplified through PCR. (ii) Similarly, an *ermB* erythromycin resistance marker was also amplified through PCR, such that target sites for the restriction enzyme *Eco*RV were added on each end. The sequences of the primers, annotated in green, are given in Table S3. (B) All three PCR products were cloned into pGEM-T Easy vectors carried by *E. coli* TOP10 cells. (iii) A blunt-ended fragment was released from the plasmid carrying the *ermB* gene through digestion with *Eco*RV. (ii) This fragment was then ligated into the plasmid carrying the region found downstream of *hexB* in the pneumococcal chromosome, pRight, after it had been digested with the blunt cutting enzyme *Bmg*BI. (C) (ii) The construct thereby generated was then amplified through PCR to add an *Eco*RV cut site onto one end and a *Not*I cut site on the other. This allowed it to be ligated into a plasmid carrying the region found upstream of *hexB* in the pneumococcal genome, pLeft, following digestion of both constructs with *Bmg*BI and *Not*I, which cuts in the multiple cloning site of pGEM-T Easy. (i) This produced a plasmid carrying the *ermB* gene flanked by the sequences found either side of *hexB* in the pneumococcal chromosome, making it suitable for knocking out the mismatch repair gene in *S. pneumoniae*.
(PDF)

Table S1 Transformation frequencies in the experiments from which sequenced isolates were taken. Three independent transformation experiments were performed at a high concentration of DNA (500 ng mL^−1^) and six independent transformation experiments were performed at a low concentration of DNA (5 ng mL^−1^). Each experiment was separately plated onto 5% horse blood agar plates supplemented with either 200 g L^−1^ kanamycin alone or both 200 g L^−1^ kanamycin and 100 mg L^−1^ ampicillin. The displayed values represent the mean CFU count, with the standard deviation given in parentheses, from the selective plates relative to the mean total CFU count from three sets of serial dilutions onto 5% horse blood agar plates containing no antibiotic selection.
(DOCX)

Table S2 Accession codes for Illumina data used in this project.
(DOC)

Table S3 Sequences of primers used in this study.
(DOC)

Text S1 The impact of sequence similarity on the positioning of secondary RSSs. This document describes a more detailed assessment of whether the level of sequence similarity between donor and recipient strains significantly affects the positioning of unselected recombinations in this experiment.
(DOCX)
