# Supplementary figures and images for: A High-Resolution View of Genome-Wide Pneumococcal Transformation
Source: PLoS Pathog. 2012 Jun 14;8(6):e1002745. doi: 10.1371/journal.ppat.1002745 (PMC3375284; doi:10.1371/journal.ppat.1002745)

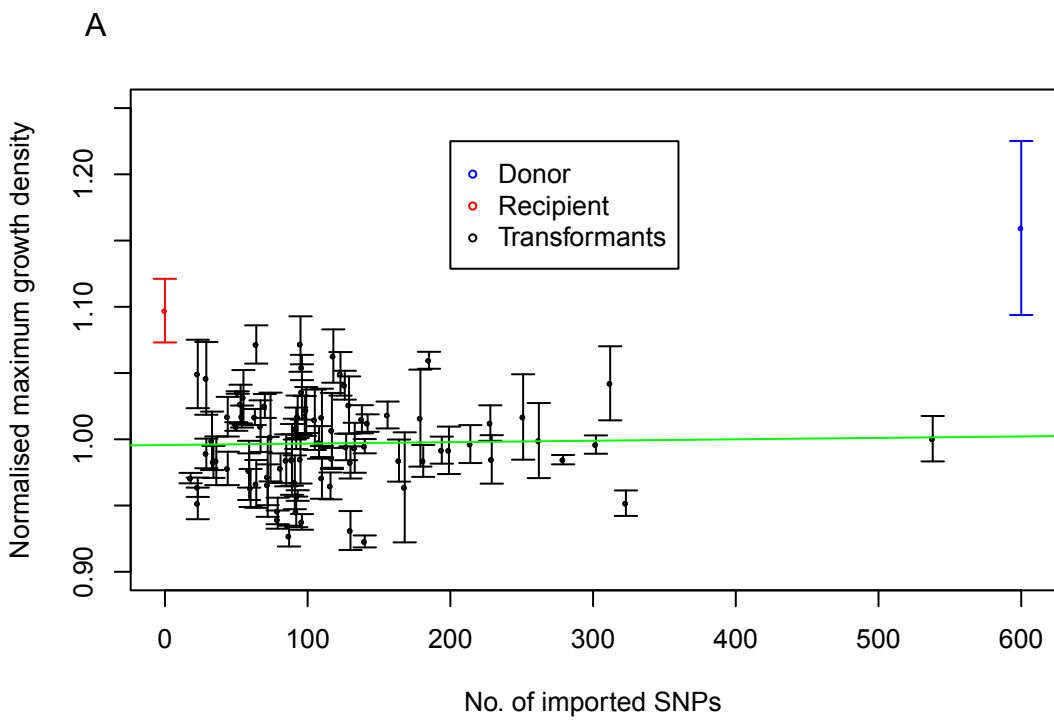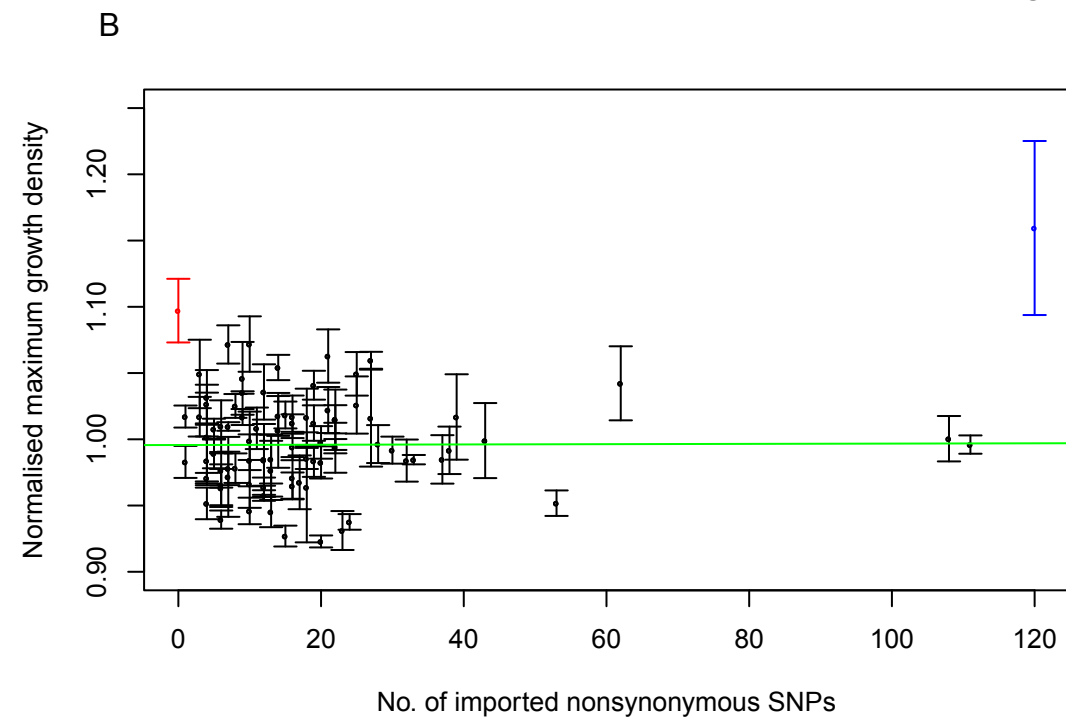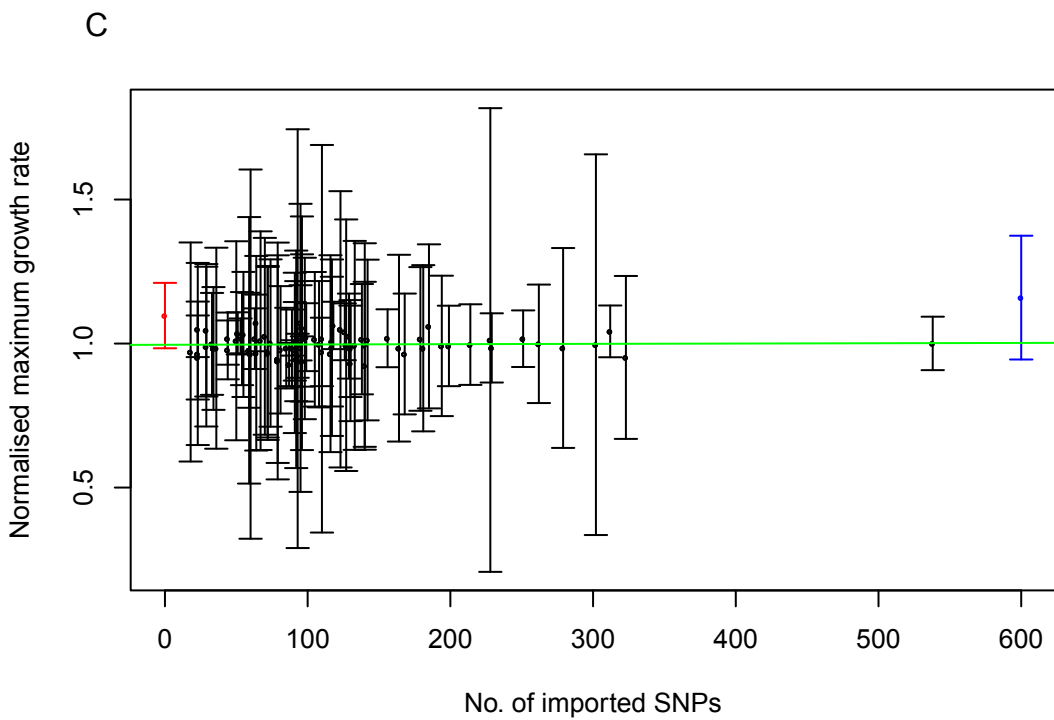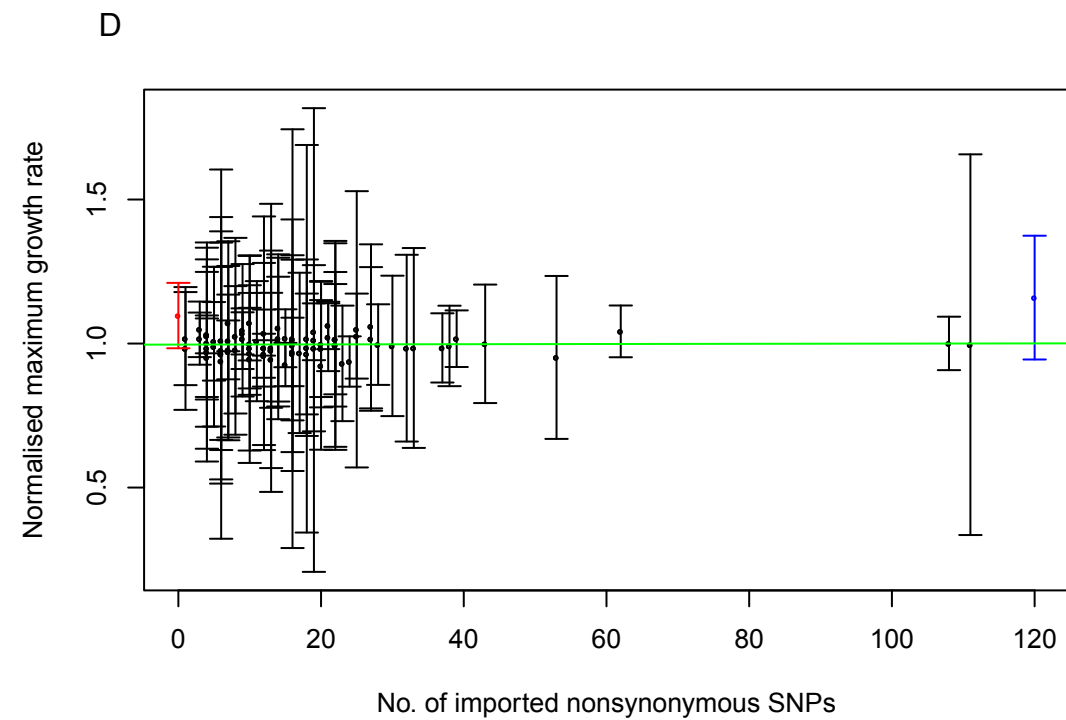

Supplement: Figure S1 — In vitro growth characteristics of transformed isolates. The displayed data points, represented as error bars encompassing one standard error of the mean above and below the mean value, summarise three independent biological replicates of growing the transformed isolates in Brain-Hearth infusion broth. Maximum growth density and rate measurements from each replicate were separately scaled relative to the mean value across all strains in the experiment to account for any systematic differences between the replicates. Data for transformants are shown in black; the corresponding points for the donor strain (TIGR4Δcps) are in blue and for the recipient strain (23F-R) in red. The maximum density to which the isolates grew is shown relative to the total number of SNPs transferred through transformation in (A), and relative to the number of non-synonymous SNPs transferred through transformation in (B). In both cases, the datapoint for the donor strain is shown at the extreme right of the x axis, although 20,773 total marker SNPs, and 5,246 nonsynonymous SNPs, actually distinguish it from the recipient. The green line shows the maximum growth density modelled as a linear function of the x axis in each case. In both cases, no significant correlation was found (Pearson correlations R 2 = 7.14×10^−4^ and 3.50×10^−4^, p values = 0.81 and 0.87, for all marker SNPs and non-synonymous marker SNPs respectively). All transformants have a lower peak growth value than both the donor and recipient strains; this appears to be the consequence of the selected recombination, as it is a trait common to all isolates. This indicates there may be an aspect of the recipient genotype that interacts detrimentally with the acapsular phenotype or the expression of kanamycin resistance. The maximum rate at which the isolates were found to grow in the same experiments is shown relative to the total number of SNPs transferred through transformation in (C), and relative to the number of non-synonymous SNPs [file ppat.1002745.s001.pdf]

## Cullen and Frey graph

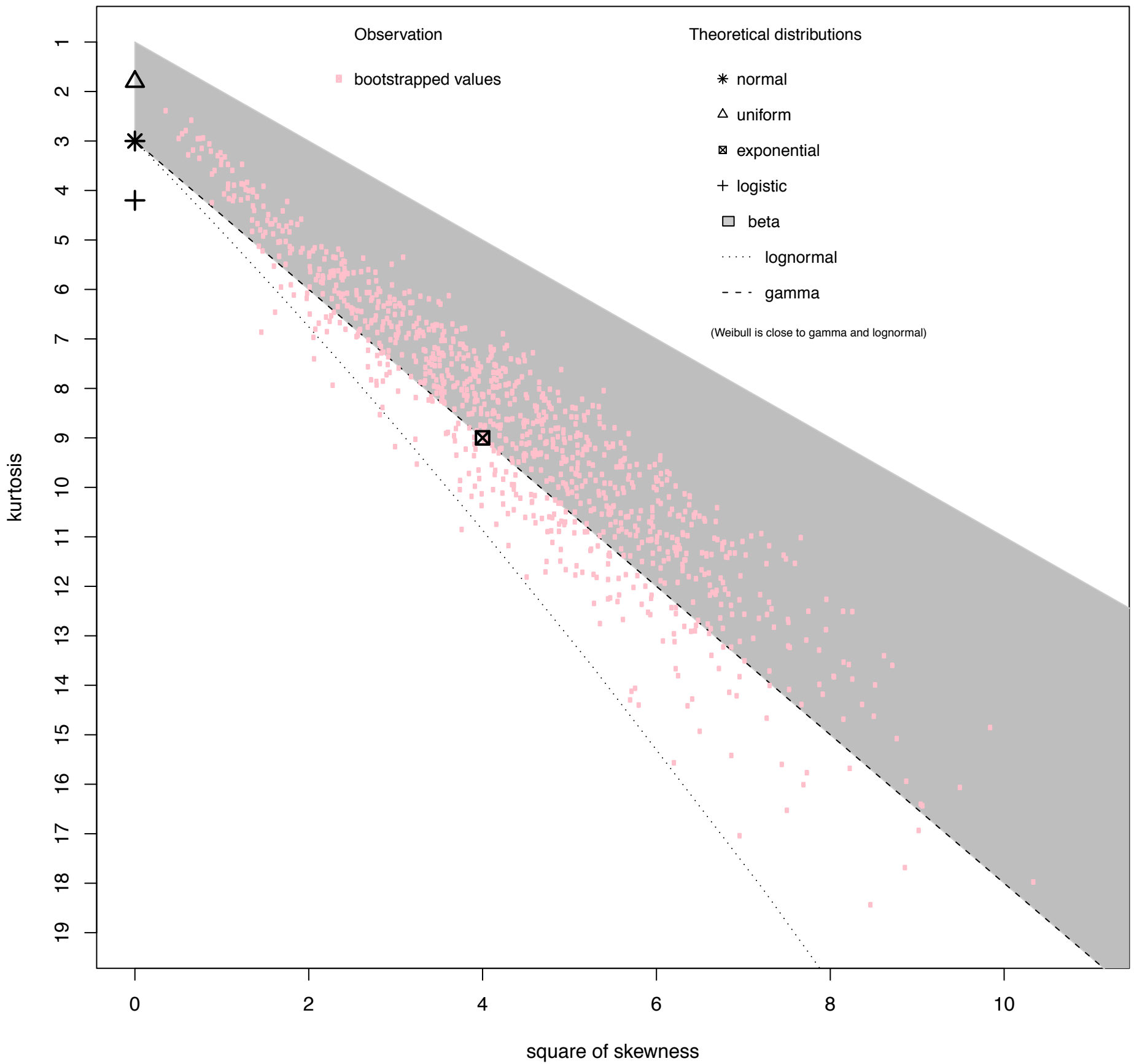

Supplement: Figure S2 — Distribution of transformation event lengths. This Cullen-Frey plot describes the skewness and kurtosis of the L50R measurements of secondary RSSs. The bold red point indicates the properties of the overall dataset; the pale pink points represent 1,000 bootstrapped replicates from the same dataset. The bootstrapped data points run along the line that suggests the data are described by a gamma distribution; their clustering suggests an exponential distribution, a specific case of the gamma distribution, is appropriate in this case. (PDF) [file ppat.1002745.s002.pdf]

**Figure S3****A**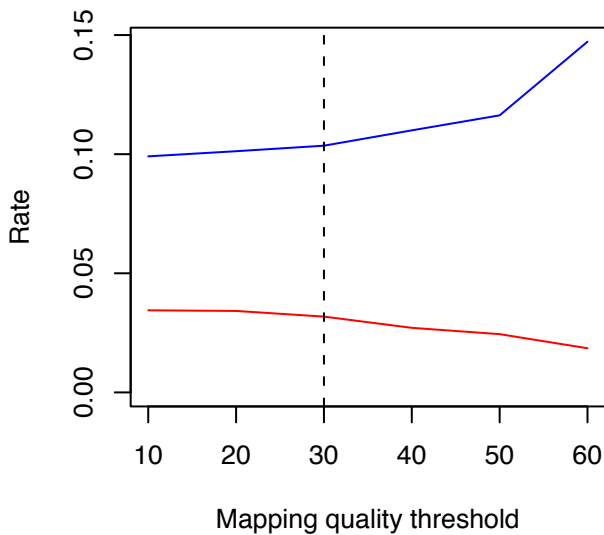**B**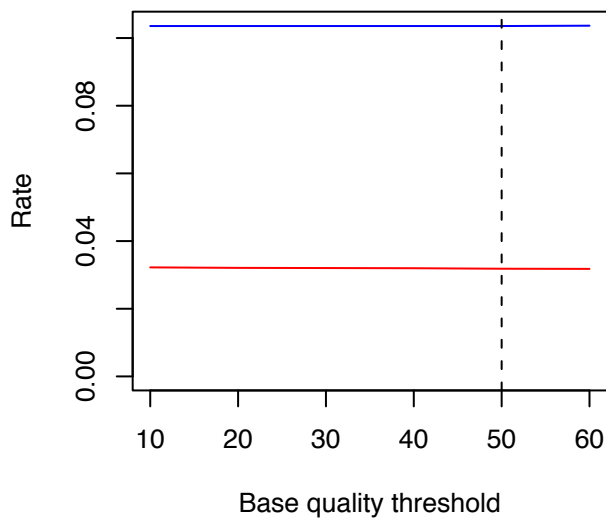**C**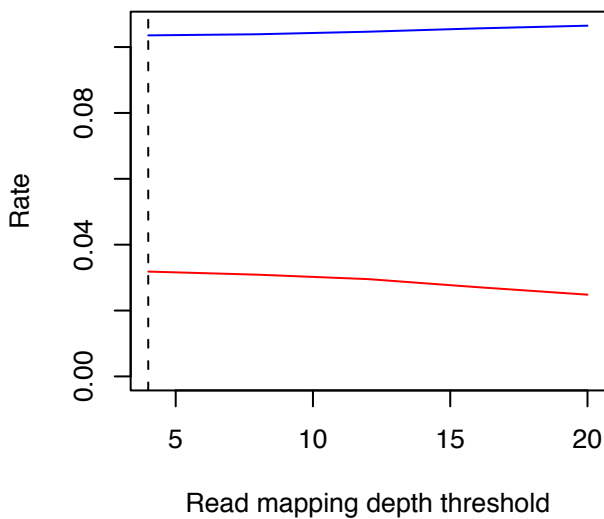**D**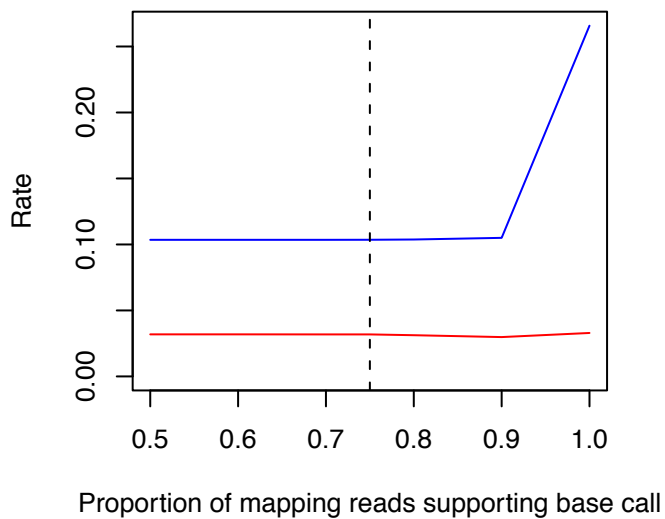

Supplement: Figure S3 — Evaluation of SNP identification parameters. These graphs plot the change in the false positive rate (red) and false negative rate (blue) with changes in the value of mapping parameters. The false positive and negative rates were assessed by comparing the set of polymorphisms identified by mapping the Illumina data from the sequencing of the donor DNA against the reference set identified through the whole genome alignment. On each plot, the parameter value used in previous publications [35], [42] is indicated by a vertical dashed line. The parameters evaluated are (A) mapping quality threshold for including a read in the mapping analysis, (B) base quality threshold for use of a read base in SNP calling, (C) minimum number of mapped sequence read bases needed to call a base, and (D) the minimum proportion of the mapped sequence reads that must support a base call at an individual reference nucleotide for the base to be identified. These data show that altering these parameters generally has relatively little impact on the sensitivity and specificity of SNP identification. It should be noted that the false positive and negative rates calculated in this evaluation correspond to that which may be expected when mapping Illumina reads from one pneumococcal isolate onto the reference chromosome from a divergent genotype. They do not reflect the rates expected when identifying SNPs in the transformant sequences, which are much more closely related to the recipient 23-F strain used as the reference genome. (PDF) [file ppat.1002745.s003.pdf]

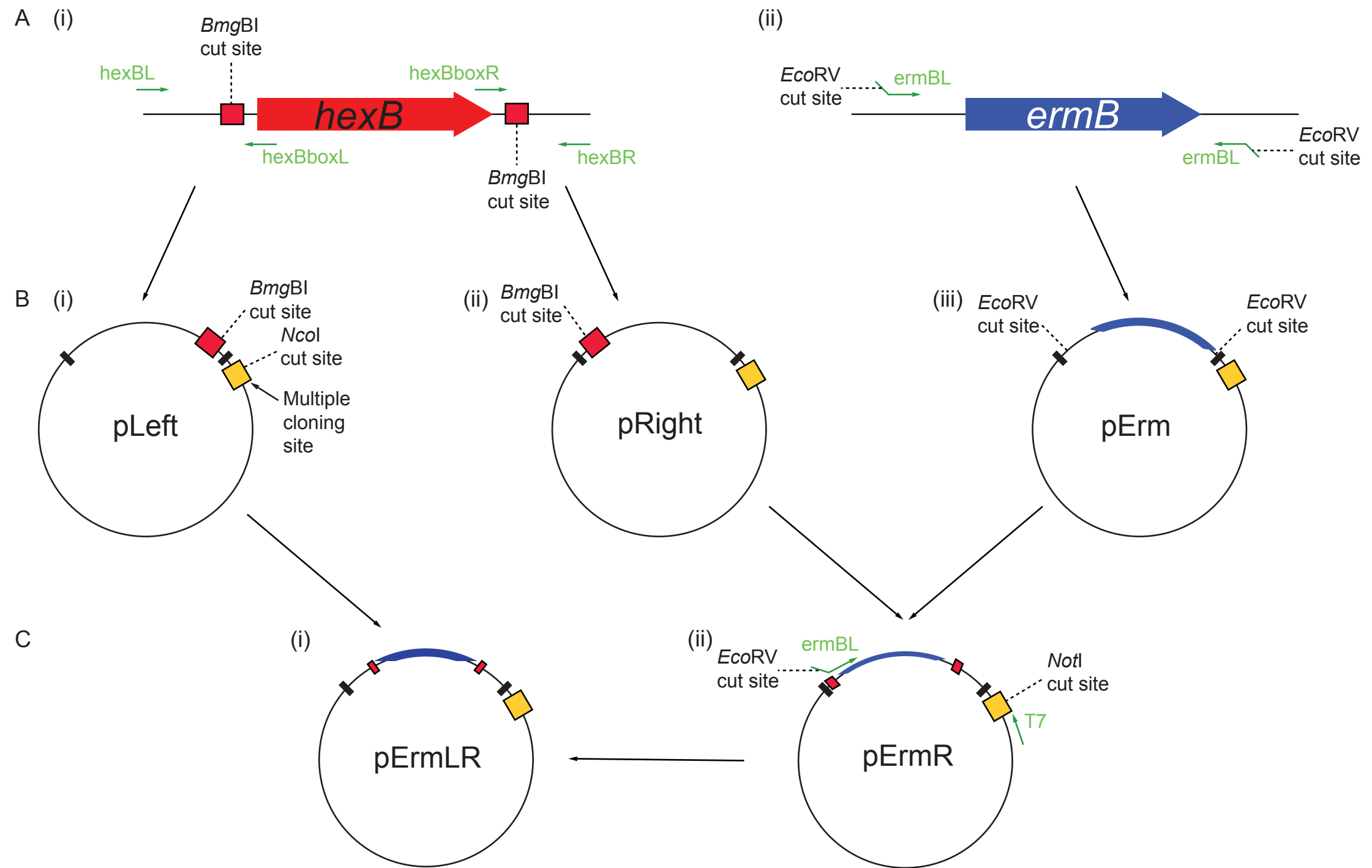

Supplement: Figure S4 — Construction of S. pneumoniae 23F-RΔhexB. (A) (i) Upstream and downstream regions flanking hexB, both containing BmgBI cut sites within BOX elements (indicated by red boxes), were amplified through PCR. (ii) Similarly, an ermB erythromycin resistance marker was also amplified through PCR, such that target sites for the restriction enzyme EcoRV were added on each end. The sequences of the primers, annotated in green, are given in Table S3. (B) All three PCR products were cloned into pGEM-T Easy vectors carried by E. coli TOP10 cells. (iii) A blunt-ended fragment was released from the plasmid carrying the ermB gene through digestion with EcoRV. (ii) This fragment was then ligated into the plasmid carrying the region found downstream of hexB in the pneumococcal chromosome, pRight, after it had been digested with the blunt cutting enzyme BmgBI. (C) (ii) The construct thereby generated was then amplified through PCR to add an EcoRV cut site onto one end and a NotI cut site on the other. This allowed it to be ligated into a plasmid carrying the region found upstream of hexB in the pneumococcal genome, pLeft, following digestion of both constructs with BmgBI and NotI, which cuts in the multiple cloning site of pGEM-T Easy. (i) This produced a plasmid carrying the ermB gene flanked by the sequences found either side of hexB in the pneumococcal chromosome, making it suitable for knocking out the mismatch repair gene in S. pneumoniae. (PDF) [file ppat.1002745.s004.pdf]
