# Supplementary material for: A High-Resolution View of Genome-Wide Pneumococcal Transformation
Source: PLoS Pathog. 2012 Jun 14;8(6):e1002745. doi: 10.1371/journal.ppat.1002745 (PMC3375284; doi:10.1371/journal.ppat.1002745)
Supplement: Table S1 — Transformation frequencies in the experiments from which sequenced isolates were taken. Three independent transformation experiments were performed at a high concentration of DNA (500 ng mL−1) and six independent transformation experiments were performed at a low concentration of DNA (5 ng mL−1). Each experiment was separately plated onto 5% horse blood agar plates supplemented with either 200 g L−1 kanamycin alone or both 200 g L−1 kanamycin and 100 mg L−1 ampicillin. The displayed values represent the mean CFU count, with the standard deviation given in parentheses, from the selective plates relative to the mean total CFU count from three sets of serial dilutions onto 5% horse blood agar plates containing no antibiotic selection. (DOCX) [file ppat.1002745.s005.docx]

| **Transformation condition** | **Frequency of kanamycin resistant transformants** | **Frequency of penicillin and kanamycin transformants** |
| --- | --- | --- |
| High [DNA] (1) | 4.15 x10^-5^ (3.58x10^-6^) | 3.98 x10^-5^ (5.47x10^-6^) |
| High [DNA] (2) | 4.41 x10^-5^ (5.49x10^-6^) | 3.75 x10^-5^ (1.58x10^-6^) |
| High [DNA] (3) | 3.68 x10^-5^ (1.87x10^-6^) | 3.22 x10^-5^ (5.54x10^-6^) |
| Low [DNA] (1) | 2.56 x10^-7^ (6.98x10^-8^) | 3.88 x10^-8^ (1.34x10^-8^) |
| Low [DNA] (2) | 4.96 x10^-7^ (5.37x10^-8^) | 2.95 x10^-7^ (2.69x10^-8^) |
| Low [DNA] (3) | 6.59 x10^-7^(1.50x10^-7^) | 6.12 x10^-7^ (9.40x10^-8^) |
| Low [DNA] (4) | 6.43 x10^-7^ (1.71x10^-7^) | 8.60 x10^-7^ (6.15x10^-8^) |
| Low [DNA] (5) | 8.76 x10^-7^ (8.17x10^-8^) | 8.22 x10^-7^ (1.88x10^-7^) |
| Low [DNA] (6) | 8.22 x10^-7^ (9.40x10^-8^) | 6.51 x10^-7^ (6.15x10^-8^) |
