# Supplementary material for: A High-Resolution View of Genome-Wide Pneumococcal Transformation
Source: PLoS Pathog. 2012 Jun 14;8(6):e1002745. doi: 10.1371/journal.ppat.1002745 (PMC3375284; doi:10.1371/journal.ppat.1002745)
Supplement: Table S3 — Sequences of primers used in this study. (DOC) [file ppat.1002745.s007.doc]

| **Primer Name** | **Primer Sequence** | **Primer Use** |
| --- | --- | --- |
| hexBL | GCAGCTGCATCGTGAAATAC | Amplify region upstream of *hexB* |
| hexBR | TAGAGGTAGCCTGGGTTCCA | Amplify region downstream of *hexB* |
| hexBboxL | GCTGACGTGGTTTGAAGAGA | Amplify region upstream of *hexB* |
| hexBboxR | CCACGTCAGTTTTATCAGTAATCTC | Amplify region downstream of *hexB* |
| ermBL | GTCATGGATATCTGGAAATAAGACTTAGAAGCAAACTT | Amplify *ermB* gene from Tn*917* |
| ermBR | GATATCTCTCCATTCCCTTTAGTAACGTGT | Amplify *ermB* gene from Tn*917* |
| T7 | TAATACGACTCACTATAGGG | Amplify MCS of pGEM-T Easy |
