# Supplementary material for: A High-Resolution View of Genome-Wide Pneumococcal Transformation
Source: PLoS Pathog. 2012 Jun 14;8(6):e1002745. doi: 10.1371/journal.ppat.1002745 (PMC3375284; doi:10.1371/journal.ppat.1002745)
Supplement: Text S1 — The impact of sequence similarity on the positioning of secondary RSSs. This document describes a more detailed assessment of whether the level of sequence similarity between donor and recipient strains significantly affects the positioning of unselected recombinations in this experiment. (DOCX) [file ppat.1002745.s008.docx]

**Text S1**

**The impact of sequence similarity on the positioning of secondary RSSs**

**Definition of sequence similarity**

In this study, sequence similarity was measured over a 100 bp window, unless otherwise specified; only recipient bases with an orthologous base in the donor were considered, such that the boundaries of the window were extended to accommodate insertions and deletions in the recipient sequence. SNP sequence similarity was calculated as the proportion of bases within half a window length on either side of the SNP that were identical in the donor and recipient genomes.

**Problems with the analysis of sequence similarity**

There are a number of biases that must be taken into account when investigating the impact of sequence identity on recombination distributions. The non-uniform distribution of SNPs between the donor and recipient sequences mean that there are large numbers of low-identity polymorphisms, and many short inter-SNP distances, concentrated in small regions of the genome. This structuring of the sequence divergence is not commensurate with the requirement of many statistical tests that observations should be independent. However, regions of high identity are also problematic, as recombinations can only be observed and analysed where it is possible to detect them through the transfer of polymorphisms.

**Walking hypergeomeric test**

A ‘walking hypergeometric test’ was used to evaluate whether sequence similarity affected the positioning of RSSs (Figure 4). The recipient genome was divided into 1,000 equally sized, non-overlapping windows, each approximately equivalent in size to the mean length of a secondary RSS. This produced 890 outside the primary locus containing a marker SNP, which varied between 81% and 100% in their mean SNP sequence identity; of these, 168 overlapped a detected secondary RSS. A threshold value was ‘walked’ from the lower bound sequence identity to the maximum and the proportion of all windows containing a marker SNP, and just those overlapping observed secondary RSSs, with a mean SNP identity of, or below, the threshold value was calculated. This allowed a hypergeometric test for enrichment of secondary RSSs in genomic regions with high levels of sequence similarity between donor and recipient to be performed at each value of the threshold where at least one window overlapped with a secondary recombination. The *p* value from this test, assuming a null hypothesis that sequence identity has no effect on the positioning of recombinations, never suggested that RSSs were significantly enriched in regions of high sequence similarity between the donor and recipient.

This relatively intuitive test is still subject to some potential biases. As many RSSs are shorter than the window length, the distribution of marker SNPs within each window will determine the likelihood of an undetectable recombination overlapping with the window. Additionally, as the windows are defined relative to the recipient’s sequence, the proportion of the locus that has homologous sequence in the donor will also impact on the likelihood of an undetectable recombination occurring within a window. Hence a sliding window approach was used to minimise these problems.

**Sliding window analysis algorithm**

Those bases in the recipient genome with orthologous bases in the donor sequence (*i.e.* the set of bases that excludes all sequence insertions in either the donor or recipient strain) can be defined as ‘core genome bases’. Each secondary recombinant segment was analysed independently; the number of core genome bases within the RSS was used as the window size (*i.e.* the L50_R_ minus insertions in the recipient), which was moved along each core genome base in the recipient sequence. Using the set of marker SNPs that could be detected by mapping Illumina reads generated from the donor DNA against the recipient strain reference sequence, at each position it was recorded as to whether the recombination could be detected or not. If it could be identified, then the total number of core genome bases within the two BRs and the mean identity of the SNPs within the RSS were recorded. This allowed the calculation of the proportion of possible recombinations containing the same number of core genome bases with the same, or greater, mean SNP identity and, separately, BRs of equal, or greater, length.

**Results of the sliding window analysis**

The two null hypotheses were that the proportions, calculated for each secondary RSS, would fit a uniform distribution in both cases; that is, neither the mean SNP identity, nor the number of core genome bases in the two BRs, would differ from that expected by chance, given the observed lengths of RSSs. To test this, the observed distribution of *p* values were subjected to a Chi squared goodness-of-fit test relative to the uniform distribution, split into ten categories. For both the mean SNP identity and the total number of core genome bases in the BRs, the *p* value assuming this null hypothesis was found to be 1.0.

**Potential biases**

One potential bias was the method used to measure SNP identity. However, the test’s outcome was found to be independent of the length of flanking sequence used to determine local sequence similarity around the SNP; using 50, 250, 500 and 1,000 bp either side gave the same *p* value. Another potential bias was the consequence of missing data at marker sites. As the sliding window analysis assumed that the allele at all marker polymorphisms could be determined, there was a risk that in some cases the BR lengths observed in the experimental data risked being biased by those RSSs where the flanking polymorphisms were ambiguous, hence boundaries were artefactually longer due to this missing data. However, excluding the three secondary RSSs where the FRs could not be defined at the highest level of precision had no effect on the calculated *p* value.

**Conclusion**

The sliding window analysis indicates that neither mean sequence identity, nor the requirement for a minimum length of perfect sequence identity at the edges of RSSs, significantly influences the positioning of unselected recombinations in this system. This is in concordance with the independent walking hypergeometric test analysis.
